# Supplementary figures and images for: Strain-Specific Biostimulant Effects of Chlorella and Chlamydomonas Green Microalgae on Medicago truncatula
Source: Plants (Basel). 2021 May 25;10(6):1060. doi: 10.3390/plants10061060 (PMC8227499; doi:10.3390/plants10061060)

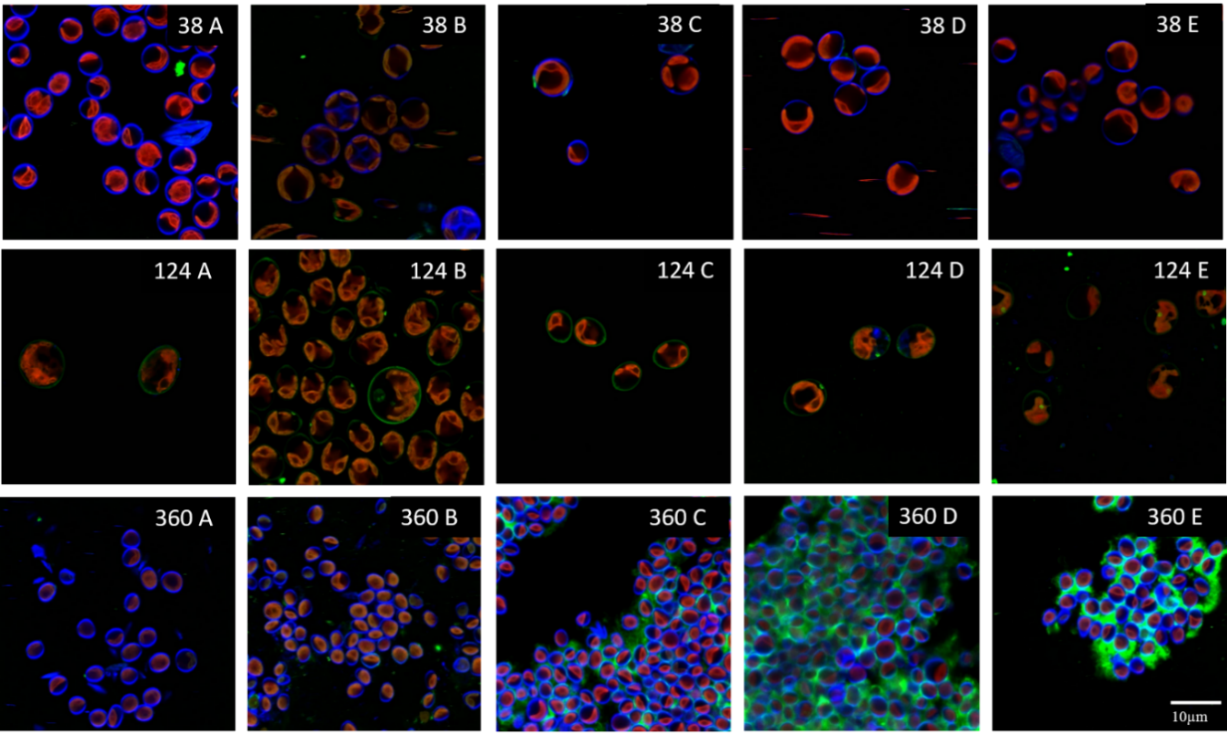

Supplement: Supplementary file 1 [file plants-10-01060-s001.zip › Fig S1.tif]
